# Strain of *Xanthomonas oryzae* pv. *oryzae* Loses Virulence through Dysregulation of Cardiolipin Synthase

**DOI:** 10.3390/plants13182576

**Published:** 2024-09-14

**Authors:** Yiqun Hu, Qingqing Chen, Aifang Zhang, Liyuan Zhang, Hansong Dong

**Affiliations:** 1 Anhui Province Key Laboratory of Pesticide Resistance Management on Grain and Vegetable Pests, Institute of Plant Protection and Agro-Products Safety, Anhui Academy of Agricultural Sciences, Hefei 230031, China; huyiqun@aaas.org.cn (Y.H.); sunnysky1987@126.com (Q.C.); 2 National Key Laboratory of Wheat Improvement, College of Plant Protection, Shandong Agricultural University, Taian 271018, China; hsdong@sdau.edu.cn

**Keywords:** sRNA trans3287, bacterial virulence, biofilm formation, pathogen-host interaction

## Abstract

Small non-coding RNAs (sRNAs) are pivotal post-transcriptional regulatory factors influencing biological activity. Studies on the rice bacterial blight pathogen *Xanthomonas oryzae* pathovar *oryzae* strain PXO99^A^, previously identified a virulence-associated sRNA, trans3287. A mutant strain lacking this sRNA, named SK01, resulted in markedly diminished virulence towards rice. This study aims to further elucidate the underlying bacterial virulent function of trans3287. The expression of trans3287 was quantified in virulence-inducing and standard nutritional conditions to clarify its production mechanism. The detection of virulence-associated genes revealed that trans3287 regulated the synthesis processes of extracellular polysaccharides, lipopolysaccharides, and the type III secretion system. Moreover, bioinformatics prediction and quantitative PCR indicated a potential direct target of trans3287, *PXO_03470*, encoding cardiolipin synthase. A dual-plasmid system fusing with GFP tag and protein immunoblotting confirmed that sRNA trans3287 negatively regulated *PXO_03470*. Bacterial biofilms demonstrated trans3287 regulated the disruption of biofilm integrity through cardiolipin synthase. This study provides preliminary insights into the mechanistic underpinnings of the role of sRNA trans3287 in mediating bacterial virulence through cardiolipin synthase.

## 1. Introduction

*Xanthomonas oryzae* pv. *oryzae*, a pathogenic variant of *X. oryzae*, infects rice (*Oryza sativa* L.) through hydathodes or wounds, colonizes the vascular system, and causes bacterial blight of rice. This disease reduces rice yield and quality, posing significant threats to rice production [1,2]. The predominant strategies for managing this disease include the use of resistant varieties and chemical treatments [3]. While breeding resistant varieties is economically viable to combat bacterial blight, the durability of monogenic resistance is challenged by rapid pathogen selection [4,5]. Moreover, long-term reliance on chemicals poses environmental and health risks and promotes resistance to pathogens, leading to treatment failures [6].

Pathogens orchestrate host interactions and virulence gene expression by integrating diverse environmental signals. Among the regulators, small non-coding RNAs (sRNAs) play a pivotal role as post-transcriptional modulators [7,8]. The fundamental regulatory function of bacterial sRNAs involves complementary pairing with target mRNAs, modifying their expression to control biological functions [9]. In animal pathogenic bacteria, the documented regulatory mechanisms of sRNAs include inhibiting translation by binding to the Shine–Dalgarno (SD) sequence, ribosomal standby sites, or translation enhancers; activating translation by disrupting the SD/AUG secondary structure; or promoting mRNA degradation through interactions with the open reading frame (ORF) [10]. Furthermore, sRNAs may also influence gene expression by directly binding to specific proteins [11]. 

In contrast to their counterparts in animal pathogens, reports on sRNAs in plant pathogenic bacteria are relatively scarce, particularly among the *Xanthomonas*. For instance, in *X. oryzae* pv. *oryzicola*, sRNA Xonc3711 binds to the coding region of *Xoc_3982*, a gene encoding a DNA-binding protein, and negatively regulates the production of *Xoc_3982* mRNA. This interaction inhibits the expression of flagellar-associated genes and biofilm formation, impacts the bacterial response to oxidative stress, and thus, regulates bacterial virulence [12]. In *X. campestris* pv. *campestris*, the sRNA RsmU was identified through genome-wide screening as a negative regulator of virulence, hypersensitivity responses, and cellular motility. Interactions between RsmU and the global post-transcriptional regulator RsmA were confirmed through electrophoretic mobility shift and co-immunoprecipitation assays. Further, Northern blot analysis revealed that RsmU exists in two isoforms, originating from the 3′-UTR of *XC1332* mRNA [13]. Transcriptomic analysis across the genome of *X. campestris* pv. *vesicatoria* identified 24 sRNAs, eight of which are related to the expression of HrpG and HrpX. Notably, sX12 and sX13 contribute to bacterial virulence, sX12 influences the interaction between *Xcc* and its host, while sX13 mediates bacterial adaptation to environmental conditions. The role of sX13 extends to regulating virulence through modulation of HrpX mRNA, and its absence affects *X. campestris* pv. *vesicatoria* virulence and the expression of type III secretion system (T3SS)-related genes. sX13 acts upstream of HrpG but does not affect HrpG mRNA accumulation. GFP reporter assays have confirmed that the protein synthesis inhibition mediated by sX13 depends on the binding of C-rich motifs in sX13 to G-rich motifs in putative target mRNAs. Intriguingly, the activity of sX13 does not rely on the chaperone protein Hfq, although the abundance of Hfq mRNA and Hfq::GFP is negatively regulated by sX13 [14]. RNAomics-based screening methods have identified four sRNAs in *Xcc*, named Xcc1, Xcc2, Xcc3, and Xcc4. Xcc1 has been highlighted to act as an integrative element, encoding a transposon and plasmid transfer intermediary sRNA, with its transcription being positively regulated by the virulence-regulating factors HrpG and HrpX [15].

The study of sRNAs in *X. oryzae* pv. *oryzae* dates back to 2011, with the identification and experimental validation of eight sRNAs, including three that depend on the Hfq protein, via genome-wide screenings [16]. Research in 2018 led to the discovery of two key virulence-related sRNAs, trans217 and trans3287, in the *X. oryzae* pv. *oryzae* strain PXO99^A^. Deletions in these sRNA sequences led to a marked decrease in pathogenicity towards host rice, hypersensitivity in non-host tobacco, and disruptions in effector protein secretion. The mechanisms underlying these observations were not deeply explored at that time [17]. Given the previous research on sRNA trans3287, the mutant strain PXO99^A^, which lacks sRNA trans3287, was designated as SK01. The current study aims to advance the target identification and regulatory function analysis of sRNA trans3287 and investigate the role of the SK01 strain in enhancing rice disease resistance.

## 2. Results

### 2.1. Regulation of sRNA trans3287 Expression in X. oryzae *pv.* oryzae under Varied Nutritional Conditions

**Figure 1:**
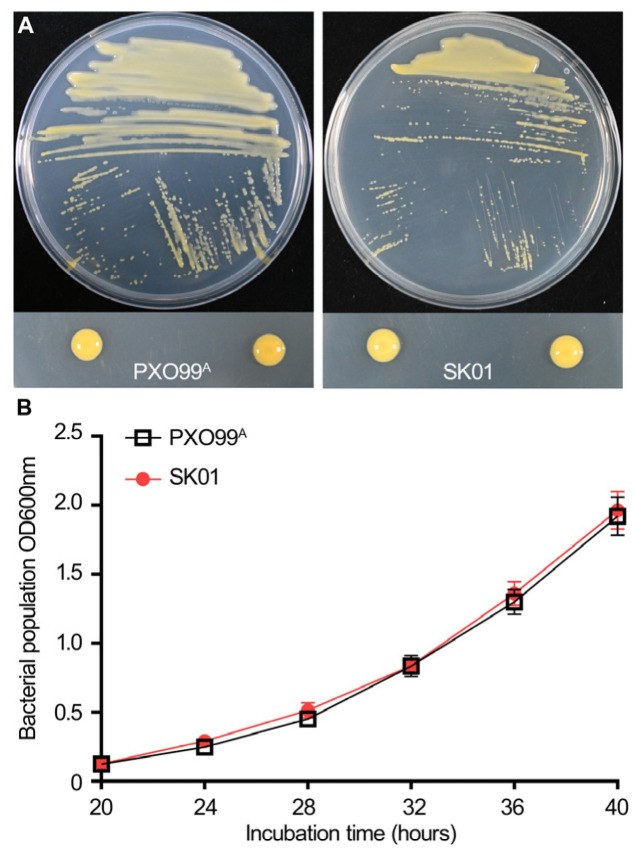
Phenotypes of strains PXO99^A^ and SK01. (**A**) The wild-type (WT) strain PXO99^A^ and sRNA-deletion mutant strain SK01 were cultured on nutrient medium for 3 days. Bacterial suspension was spread (**up**) and dropped (**below**) in plates. (**B**) Measurement of the bacterial growth rate of *Xoo* strains PXO99^A^ and SK01. The OD600 values represent bacterial cell density. Data at each time point are presented as mean ± SD (*n* = 5).

**Figure 2:**
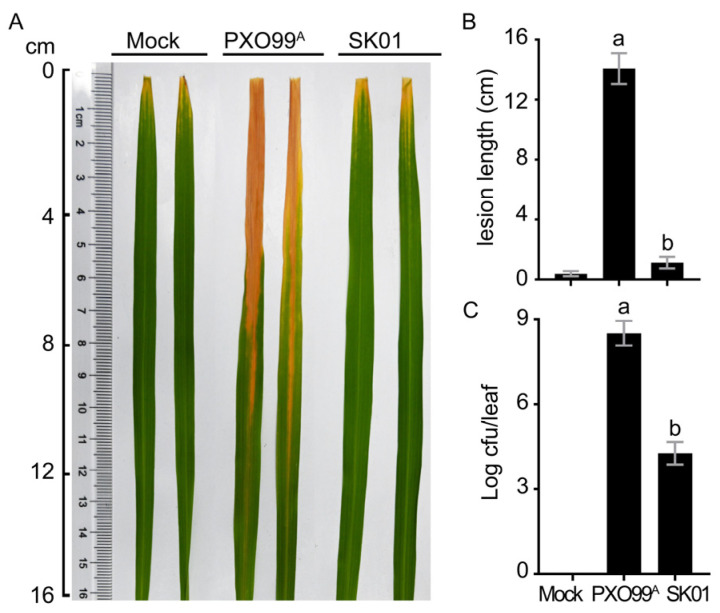
Virulence assessments of strains PXO99^A^ and SK01. PXO99^A^- and SK01-inoculated Nipponbare leaves. (**A**) Bacterial blight symptoms photographed at day 14 after leaf-top-clipping inoculations. (**B**) Blight lesion length on leaves. (**C**) Bacterial populations in Nipponbare leaves 3 days after leaf-center-infiltrating inoculations. Data shown are mean values ± SD bars (*n* = 10 leaves in (**B**); *n* = 3 experimental replicates in (**C**)). Different letters in lowercase indicate significant differences by analysis of variation using Fisher’s least significant difference test (*p* < 0.01).

**Figure 3:**
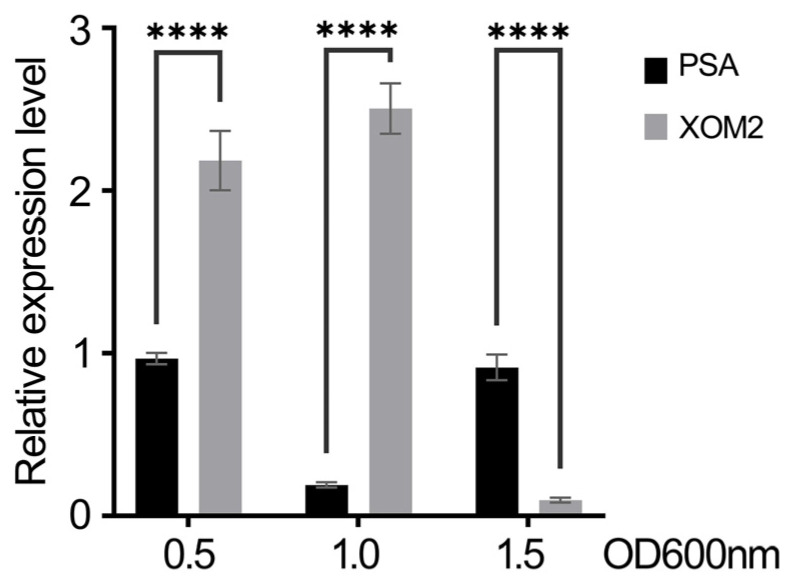
Expression levels of sRNA trans3287 in PSA and XOM2 media. *X. oryzae* pv. *oryzae* strain PXO99^A^ was cultured in PSA and XOM2 media. RNA was extracted from bacterial cultures collected at three growth phases (OD 600 nm values of 0.5, 1.0, and 1.5), and subsequently, reverse transcribed. The expression of sRNA trans3287 was measured using reverse transcriptase quantitative polymerase chain reaction (RT-qPCR), with its expression at OD 600 nm 0.5 in PSA normalized to 1. This normalization facilitated the comparison of expression levels across different time points and media, highlighting the adaptive expression behavior of sRNA trans3287 under varied culturing conditions. Data were analyzed using 2-way analysis of variance (ANOVA) followed by Tukey’s test. Four asterisks represent significant differences (*p* < 0.0001).

The sRNA trans3287 contained 455 nt and was located from 5,086,294 to 5,086,749 in the *Xoo* PXO99^A^ genome. The sequence of trans3287 was found to be highly conservative within the genus by comparing with the NCBI database. sRNA trans3287 deletion did not affect bacterial multiplication on the growth medium (Figure 1), and significantly reduced bacterial blight symptoms (Figure 2A,B) and the bacterial population (Figure 2C). The expression of sRNA trans3287 was investigated under nutrient-rich peptone sucrose agar (PSA) and nutrient-deficient XOM2 conditions, which simulated normal growth and virulence induction, respectively. Cultures of PXO99^A^ were grown in these media, and bacterial cells were collected at three growth stages (optical density [OD] 600 nm of 0.5, 1.0, and 1.5) for RNA extraction. Expression levels of sRNA trans3287 were normalized to 1 at OD 600 nm 0.5 in the PSA medium for comparative analysis. In XOM2, the expression profile of trans3287 displayed a pattern of initial stability followed by a sharp decline as the growth cycle progressed, indicating a response to the minimal nutrients in the medium. Conversely, in the nutrient-rich PSA medium, there was a notable initial decrease in expression, which subsequently rose, illustrating an adaptive expression response to environmental conditions (Figure 3). These differential expression trends underscore the significant impact of culturing conditions on the regulatory roles of sRNA trans3287, with notably higher expression under virulence-inducing conditions (before OD 600 nm reached 1.0) compared to normal growth conditions during the early growth phase. The findings imply enhanced regulatory activity of sRNA trans3287 on bacterial functions under virulence-inducing conditions during the early growth phases.

### 2.2. Downregulation of Virulence-Related Genes in X. oryzae *pv.* oryzae Strain SK01

The virulence of bacteria involves a complex interplay of multiple pathogenic factors, including extracellular polysaccharides (EPSs), lipopolysaccharides (LPSs), adhesins, biofilms, and components of the T3SS [18,19,20]. The T3SS is primarily encoded by the *hrp* gene cluster. Of note, *hrpG* serves as an upstream regulatory factor in the hrp gene regulatory cascade, playing a critical role in the virulent behaviors of the bacterium [21,22]. Key synthetic genes for EPSs and LPSs include *rpfC*, *cysB*, *metB2*, and the *wxo* family genes [23,24]. Additionally, *XadA* and *XadB* are crucial for the regulation of normal secretion of bacterial adhesins [25,26].

Previous findings established that deletion of sRNA trans3287 resulted in a substantial reduction of pathogenicity towards host rice. To explore whether this effect involves modulation of specific pathogenic factors, the expression impact of sRNA trans3287 deletion was examined on a set of virulence-related genes, including *rpfC*, *cysB*, *metB2*, *wxoA*, *wxoB*, *wxoD*, *XadA*, *XadB*, and *hrpG*.

**Figure 4:**
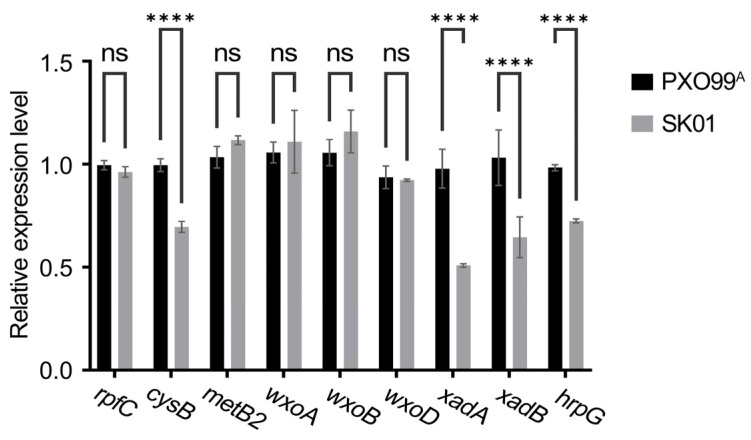
Expression levels of key virulence-related genes in *Xoo* under virulence-inducing conditions. *X. oryzae* pv. *oryzae* strain PXO99^A^ was cultured in XOM2, a medium that induces virulence, until reaching an OD 600 nm of 1.0. The bacterial cells were collected for RNA extraction and reverse transcription. The expression levels of key virulence-related genes *rpfC*, *cysB*, *metB2*, *wxoA*, *wxoB*, *wxoD*, *XadA*, *XadB*, and *hrpG* were assessed using RT-qPCR. Data were analyzed using 2-way analysis of variance (ANOVA) followed by Tukey’s test. “ns” stands for no significant difference. Four asterisks represent significant differences (*p* < 0.0001).

Cultures of the wild-type and SK01 strains were grown in XOM2 medium to an OD 600 nm of 1.0, followed by RNA extraction from the collected bacterial cells. Quantitative PCR was used to analyze the expression of virulence-related genes. The findings indicated a significant downregulation in the expression levels of *cysB*, *XadA*, *XadB*, and *hrpG* genes in the sRNA trans3287-deletion mutant strain compared to the wild-type strain (as shown in Figure 4). Other genes did not exhibit substantial expression changes, highlighting that the specific regulatory role of sRNA trans3287 is the synthesis processes of bacterial EPSs, LPSs, and T3SS.

### 2.3. Prediction of Target Genes for sRNA trans3287 in X. oryzae *pv.* oryzae

**Figure 5:**
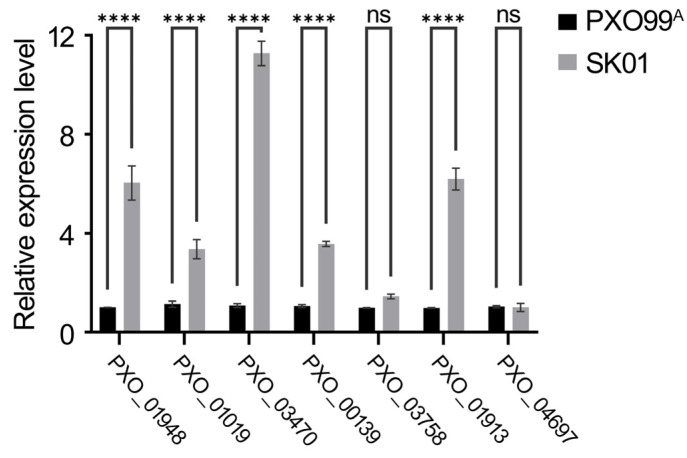
RT-qPCR analysis of the predicted target genes of sRNA trans3287. TargetRNA3 (https://cs.wellesley.edu/~btjaden/TargetRNA3/, accessed on 10 October 2021) predicted candidate target genes of sRNA trans3287 in the PXO99^A^ genome. PXO99^A^ and SK01 were cultured in XOM2 medium until reaching an OD 600 nm of 1.0. Bacterial cells were collected, RNA was extracted and reverse transcribed, and RT-qPCR was performed to measure the expression levels of the candidate targets. Data were analyzed using 2-way analysis of variance (ANOVA) followed by Tukey’s test. Four asterisks represent significant differences (*p* < 0.0001). “ns” stands for no significant difference.

Research into the functional role of bacterial sRNA functions progresses based on the identification of their targets. Due to the longer length of sRNAs and the shorter, non-continuous sRNA–target pairing regions, pinpointing precise sRNA–target interaction sites poses a challenge. Conventional methods for target identification include bioinformatics-based predictions supplemented by experimental validation. The TargetRNA3 online platform (https://cs.wellesley.edu/~btjaden/TargetRNA3/, accessed on 10 October 2021) facilitates efficient and accurate identification of bacterial sRNA targets based on the complementary features of sRNA sequences and putative target mRNA sequences [27]. After inputting the sRNA trans3287 sequence and the PXO99^A^ genome information into the platform, candidate target genes were predicted, including those encoding alpha/beta hydrolase, c-di-GMP phosphodiesterase, cardiolipin synthase (cls), endolysin, and FMN reductase (Table 1). Subsequently, an RT-qPCR assay was used to test the potential regulatory relationship between sRNA trans3287 and the candidate target genes. The results revealed an increase, to some extent, in the expression levels of the candidate targets following the deletion of sRNA trans3287. Notably, the expression level of the CLS-encoding gene *PXO_03470* exhibited significant upregulation (Figure 5), suggesting that *PXO_03470* may be a putative functional target of sRNA trans3287.

**Figure 6:**
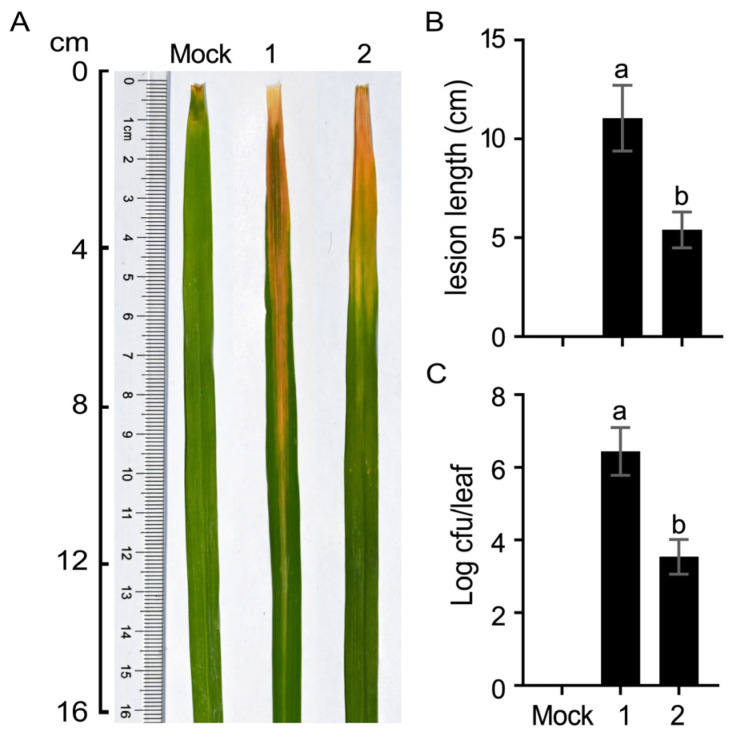
Virulence assessments of strains PXO99^A^ (1) and *ΔPXO_03470* (2) stains. *Xoo* culture-inoculated Nipponbare leaves. (**A**) Bacterial blight symptoms photographed at day 14 after leaf-top-clipping inoculations. (**B**) Blight lesion length on leaves. (**C**) Bacterial populations in Nipponbare leaves 3 days after leaf-center-infiltrating inoculations. Data shown are mean values ± SD bars (*n* = 10 leaves in (**B**); *n* = 3 experimental replicates in (**C**)). Different letters in lowercase indicate significant differences by analysis of variation using Fisher’s least significant difference test (*p* < 0.01).

The sRNA trans3287 is an important factor in bacterial virulence; so, is *PXO_03470* also involved in bacterial virulence? Based on this, we constructed the *PXO_03470*-deletion mutant strain (*ΔPXO_03470*) and tested its pathogenicity on rice. Compared with the wild-type strain PXO99^A^, the lesion length of the *ΔPXO_03470* culture decreased significantly, indicating that *PXO_03470* is essential for the virulence (Figure 6).

### 2.4. Validation of the Interaction between sRNA trans3287 and Its Target PXO_03470

**Figure 7:**
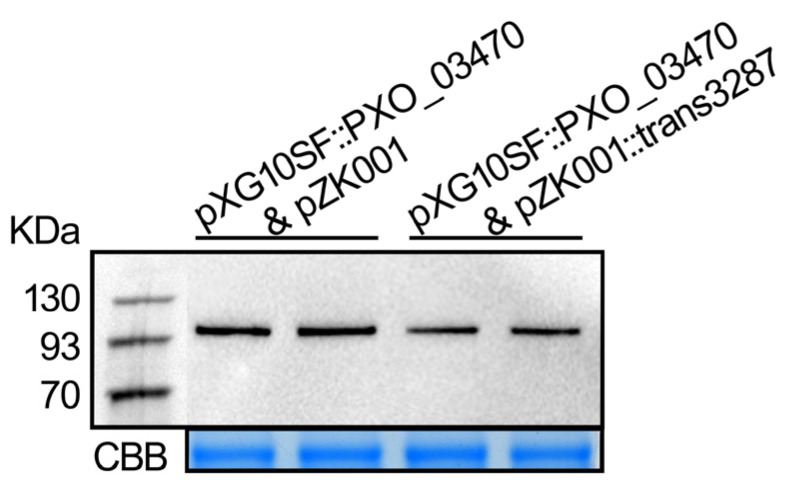
sRNA trans3287 negatively regulated the expression of CLS in protein level. The low-copy-number vector pXG10SF fused the CLS-encoding gene *PXO_03470* sequence and high-copy-number plasmid pZK001 containing the trans3287 sequence, co-transforming them into *E. coli* Top10 competent cells. Bacterial cells were collected to extract proteins for western blot analysis with monoclonal a-GFP antibodies (**upper** panel). Gel stained by Coomassie brilliant blue (CBB) as loading control (**lower** panel). The experiment was conducted with three biological replicates.

To further validate the interaction between sRNA trans3287 and the *PXO_03470* gene, a dual-plasmid system incorporating the GFP reporter gene was utilized in this study. The sRNA sequence was integrated into the low-copy-number vector pXG10SF while the candidate target sequence was incorporated into the high-copy-number vector pZK001, utilizing homologous recombination techniques. The successfully constructed recombinant vectors were co-transformed into an *Escherichia coli* TOP10 strain. Moreover, total proteins were extracted from the co-transformed strains after culture, and the GFP expression levels were assessed using western blot analysis to indicate the regulatory interaction between sRNA trans3287 and the candidate target. All samples were hybridized to show a protein band larger than 93 kD, which corresponded to the size of the fusion protein. Compared to the control group of independent plasmid pZK001, the plasmid fused with trans3287 displayed a significant reduction in GFP protein levels (Figure 7). That indicated a direct interaction between trans3287 and the CLS-encoding gene *PXO_03470*. Moreover, trans3287 negatively regulated the expression of *PXO_03470* at translational levels.

### 2.5. Impact of sRNA trans3287-Mediated Regulation of CLS on Biofilm Formation in X. oryzae *pv.* oryzae

**Figure 8:**
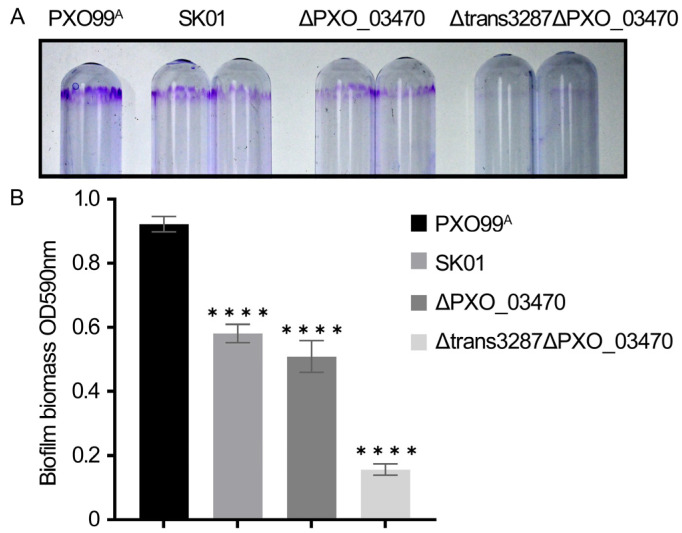
The sRNA trans3287 regulated biofilm formation by mediating CLS in *X. oryzae* pv. *oryzae*. Biofilm formation was evaluated in the wild-type strain PXO99^A^ and mutant strains *Δtrans3287*, *ΔPXO_03470*, and *Δtrans3287ΔPXO_03470* of *X. oryzae* pv. *oryzae*. (**A**) Fresh bacterial suspension was allowed to settle in glass tubes for 5 days. After the medium was discarded, tubes were washed three times with sterile water, dried at 60 °C, and stained with 1% crystal violet for 10 min. Excessive staining solution was discarded, and the tubes were rinsed three times with sterile water and dried at 37 °C before being photographed. (**B**) The stained biofilm was solubilized with 95% ethanol and mixed thoroughly through pipetting. Absorbance at 590 nm was measured using a spectrophotometer. Four asterisks represent significant differences (*p* < 0.0001).

The membranes of *X. oryzae* pv. *oryzae* are predominantly rich in cardiolipin (CL), a lipid synthesized by various CLSs, along with phosphatidylglycerol and phosphatidylethanolamine [28,29]. This study probed the biological implications of the interaction between sRNA trans3287 and the CLS-encoding gene *PXO_03470*, focusing specifically on the role of bacterial biofilm. Mutant strains lacking *PXO_03470* (*ΔPXO_03470*) and both trans3287 and *PXO_03470* (*Δtrans3287ΔPXO_03470*) were constructed to assess the biofilm phenotypes of the wild-type and mutant strains. The data indicated that deletion of both sRNA trans3287 and *PXO_03470* led to a reduction in biofilm content compared to the wild-type, with the most pronounced reduction observed in the double-deletion mutants (Figure 8). These findings demonstrated that sRNA trans3287 in *X. oryzae* pv. *oryzae* modulated the expression of target CLS and influenced biofilm formation, thereby regulating the virulence of the bacterium.

### 2.6. Enhancement of Disease Resistance in Rice by X. oryzae *pv.* oryzae Strain SK01

**Figure 9:**
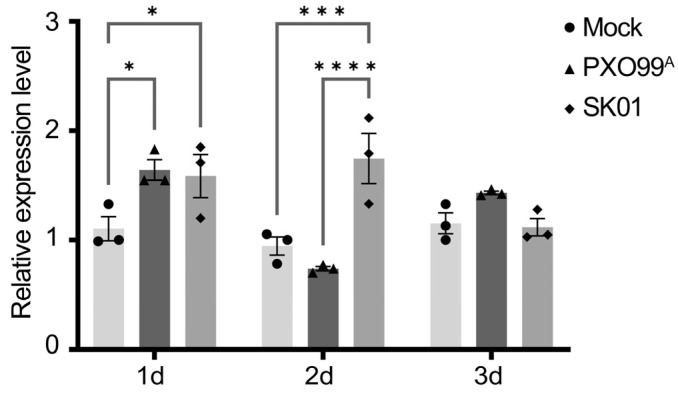
SK01 induced higher expression of rice defense response gene. Samples were collected from rice plants 24 h and 48 h after inoculation with wild-type PXO99^A^ and the mutant strain SK01. Rice RNA was extracted and reverse transcribed, followed by qPCR to measure the expression levels of the rice defense response gene *OsPR1b*. Data were analyzed using 2-way analysis of variance (ANOVA) followed by Tukey’s test. One asterisks represent significant differences (*p <* 0.05). Three asterisks represent significant differences (*p <* 0.001). Four asterisks represent significant differences (*p* < 0.0001).

The array of experiments underscored the significant contribution of sRNA trans3287 to the virulence of *X. oryzae* pv. *oryzae*, and the regulation between sRNA trans3287 and its target CLS is vital for maintaining bacterial biofilm homeostasis. In the context of plant–bacterial interactions, an inquiry was raised about the potential influence of sRNA trans3287 on the defense mechanisms of the host. To address this, an inoculation study was performed where rice plants were inoculated with the wild-type strain PXO99^A^ or the sRNA trans3287-deletion mutant strain SK01. The expression of the rice defense response gene *OsPR1b* was quantitatively measured at 24 and 48 h post-inoculation. The observations uncovered that, relative to the mock control containing only nutrient agar (NA) medium, the wild-type strain initially induced an upregulation of the defense response gene in the short term (within 24 h), followed by a notable decline around 48 h. In contrast, the SK01 strain maintained higher expression levels of the defense response gene within the first 2 days post-inoculation (24 h and 48 h) (Figure 9). These observations implied that, compared to the wild-type strain, the SK01 strain induced a prolonged defense response in rice over an extended period.

**Figure 10:**
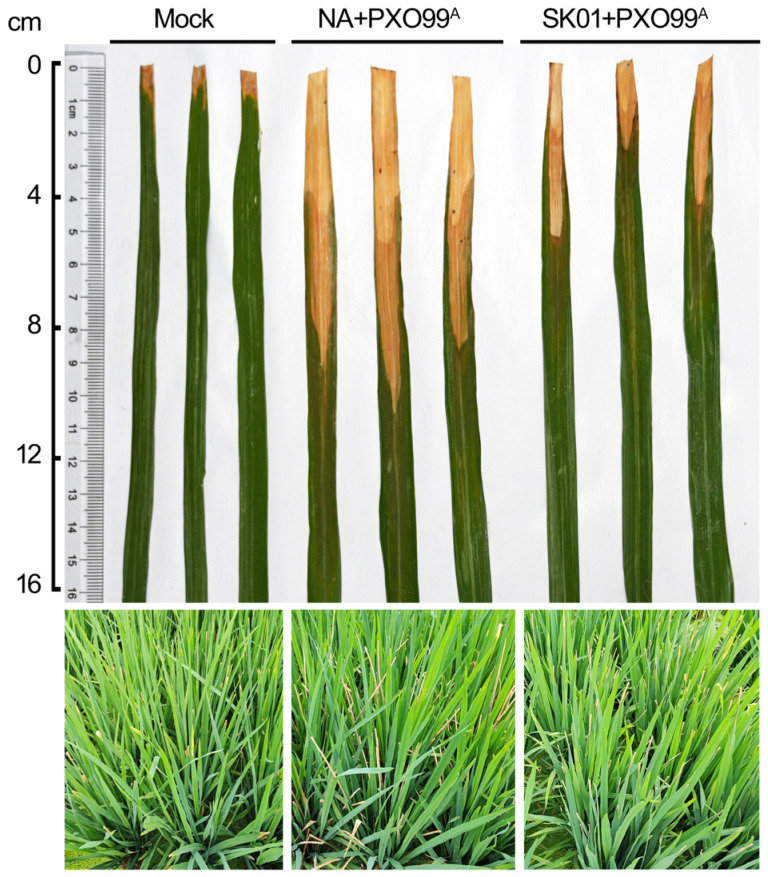
The pre-inoculation of SK01 alleviated bacterial blight symptom. The impact of pre-inoculation with PXO99^A^ and SK01 on *Xoo* severity was assessed. One-month-old seedlings of a susceptible rice variety were inoculated using sterile scissors dipped in SK01 bacterial culture (OD 600 nm = 0.5) or sterile medium as a control. Leaves were cut approximately 2 cm from the tip to introduce the bacterial fluid into the rice tissue. The plants were inoculated with PXO99^A^ 3 days later. Lesion lengths were measured 2 weeks after the second inoculation. Each group involved the inoculation of ten leaves. The experiment was conducted with three biological replicates.

The initial three days following the inoculation of rice with *X. oryzae* pv. *oryzae* are critical for the establishment of the infection. The effective colonization in the intercellular spaces of rice plants is a prerequisite for subsequent infection. Concurrently, the elevated expression of plant defense response genes can hinder the colonization process of the pathogen. This context prompted an investigation into whether an immune response initiated by pre-inoculation with the SK01 strain would influence subsequent infections. In the experimental setup, 1-month-old susceptible rice seedlings were pre-inoculated with the SK01 strain or NA medium, followed by a challenge with the virulent PXO99^A^ strain 3 days later. Disease lesions were assessed 2 weeks after the second inoculation. It was noted that rice leaves in the NA pre-inoculated group exhibited severe wrinkling and yellow-white lesions extending beyond half of the leaf along the veins. Similar symptoms were observed in the SK01 pre-inoculated group, but the degree of leaf wrinkling was less severe, and the length of the lesions was significantly reduced (Figure 10). These observations suggested that the SK01 strain triggered a more enduring defense response in rice, thereby enhancing its resistance to *Xoo* to a certain extent.

## 3. Discussion

In prior work, our team successfully identified an sRNA sequence, trans3287, in the *X. oryzae* pv. *oryzae* strain PXO99^A^ through transcriptome sequencing coupled with phenotypic experiments. The deletion of this sRNA resulted in a near-complete loss of pathogenicity on host rice [17]. The present study further explored the function and regulation mechanisms of sRNA trans3287. The investigations revealed that trans3287 exhibited different expression patterns under various culturing conditions (nutrient-rich and nutrient-deficient conditions), with significantly higher expression levels under virulence-inducing conditions (XOM2 medium) during the early growth stages of the bacteria compared to under normal conditions (PSA medium). Importantly, the regulation of trans3287 is complex and influenced not only by the bacterial culturing conditions but also by the bacterial growth phase. Further analysis demonstrated that the sRNA trans3287-deletion mutant had markedly diminished expression of key virulence-related genes, such as *cysB*, *XadA*, and *hrpG*. Moreover, sRNA trans3287 played a regulatory role in critical pathogenic processes, such as the secretion of bacterial EPSs and adhesins, as well as the T3SS. The interaction targets of sRNA trans3287 were predicted using an online prediction tool followed by experimental validation through a dual-plasmid system with a *gfp* reporter gene, confirming a direct interaction and negative regulatory effect of trans3287 on the CLS-encoding gene *PXO_03470*. Subsequent biofilm phenotype experiments elucidated that sRNA trans3287 was crucial for maintaining bacterial biofilm homeostasis via its regulation of the target CLS and biofilm formation. These findings enhance our understanding of the molecular underpinnings of bacterial pathogenicity mediated by sRNA trans3287.

It is significant to remember that this study has certain limitations. For instance, variations in computation techniques may result in inadequate predictions when bioinformatics forecasts sRNA regulatory targets. Apart from *PXO_03470*, there could be additional regulatory targets. This study reveals only one branch of the sRNA trans3287 regulatory network. The systematic approach of the sRNA research presented here can be used as a reference. The research integrates high-throughput sequencing with experimental approaches to dissect the regulatory role of sRNA trans3287 in *X. oryzae* pv. *oryzae*. This approach not only deepened our insights into the sRNA-mediated regulatory networks involved in pathogenicity but also established a foundational methodology for the identification and functional elucidation of sRNAs in *X. oryzae* pv. *oryzae*. This research enhances our understanding of bacterial virulence and provides a reference for future sRNA research into plant pathogens.

After preliminary elucidation of the regulatory mechanism of sRNA trans3287, efforts were made to explore its broader applications. The inoculation of *X. oryzae* pv. *oryzae*-susceptible rice varieties with the sRNA trans3287-deletion mutant SK01 resulted in a sustained high expression of defense response genes, suggesting that SK01 could effectively stimulate the rice immune response. When these pre-inoculated rice plants were subsequently challenged with a virulent strain, the SK01 pre-inoculated group displayed noticeably milder disease symptoms. This finding showed that pre-inoculation with the non-virulent strain SK01 before inoculation with virulent strains fostered the resistance of rice to subsequent infections by virulent strains. Notably, these results corroborated that the stimulated rice immune response induced by SK01 could substantially improve the host’s disease resistance. This insight could inspire a strategy for immune stimulation against *X. oryzae* pv. *oryzae* using non-virulent *X. oryzae* pv. *oryzae* strains to elicit a vaccine-like effect in plants, thereby boosting resistance to *X. oryzae* pv. *oryzae*. Also, risk assessment cannot be ignored. This strategy should be tested on more rice varieties and monitored for the entire growth period of rice. This approach offers a promising new direction for the management and control of *X. oryzae* pv. *oryzae* in rice.

However, further research is needed to fully understand the long-term efficacy and potential ecological impacts of this approach. Future studies should also explore the broader applicability of this strategy across different rice varieties and other crops affected by bacterial pathogens. Moreover, integrating these findings with advanced genomic and biotechnological tools could further refine and optimize plant immune responses, offering new avenues for combating a range of plant diseases.

## 4. Materials and Methods

### 4.1. Strains and Medias Informationon

The wild-type *Xoo* strain PXO99^A^ and different sRNA-related strains are maintained in the lab of National Key Laboratory of Crop Biology, Shandong Agricultural University. The two *E. coli* strains (a TOP10 strain: containing plasmids pXG10SF; a Mach1-T1 strain: containing the plasmids pZK001) were supplied by Professor Zhengfei Liu (State Key Laboratory of Agricultural Microbiology and Hongshan Laboratory, College of Veterinary Medicine, Huazhong Agricultural University).

Nutrient broth agar (NA) medium contains 3 g of beef extract (ThermoFisher, Waltham, MA, USA), 1 g of yeast extract (Oxoid, Basingstoke, UK), 5 g of polypeptone (Oxoid, Basingstoke, UK), 10 g of sucrose (Aladdin, Shanghai, China), and 18 g of agar (Aladdin, Shanghai, China) per liter (pH 7.0). LB medium contains 10 g of polypeptone, 5 g of yeast extract, 10 g of NaCl (Aladdin, Shanghai, China), and 18 g of agar per liter (pH 7.0). Nutrient-rich peptone sucrose agar (PSA) medium contains 10 g of polypeptone, 10 g of sucrose, and 1 g of Na-glutamate (Aladdin, Shanghai, China) per liter (pH 6.5). *X. oryzae* recipe 2 (XOM2) medium contains 1.8 g of D-xylose (Aladdin, Shanghai, China), 0.1 g of D,L-methionine (Aladdin, Shanghai, China), 1.87 g of sodium L-glutamate (Aladdin, Shanghai, China), 240 µM NaFe^3+^-EDTA (Aladdin, Shanghai, China), 5 mM MgCl_2_ (Aladdin, Shanghai, China), 14.7 mM KH_2_PO_4_ (Aladdin, Shanghai, China), and 40 µM MnSO_4_ (Aladdin, Shanghai, China) per liter (pH 6.5).

### 4.2. Construction of the X. oryzae *pv.* oryzae Mutant Strain SK01

The strain PXO99^A^ was used to construct mutants in this study. The trans3287 sequence was knocked out through double homologous recombination. Fragments of 454 bp upstream and 484 bp downstream of the knockout region were amplified by PCR. The upstream fragment was enzymatically treated with *BamH*I (Thermo Fisher, Waltham, MA, USA, ER0055) and *Hind*III (Thermo Fisher, ER0505), while the downstream fragment was enzymatically treated with *Hind*III and *Xba*I (Thermo Fisher, ER0683). Both fragments and the suicide vector pK18mobSacB, previously digested with *BamH*I and *Xba*I, were ligated using T4 ligase (Thermo Fisher, 15224017). The ligation product was introduced into competent *E. coli* cells via heat shock transformation and incubated at 37 °C for 1 day to obtain colonies. Monoclonal colonies were verified through colony PCR. Correctly sequenced monoclonal cultures were subjected to shaking incubation for plasmid extraction. Plasmids underwent double enzyme digestion to confirm correct assembly and were stored. The first homologous recombination was achieved using the pK18mobSacB vector, incorporating the sucrose-sensitive gene *SacB* and the kanamycin resistance gene *Kn*, to select for single crossovers. The recombinant plasmid was electroporated into PXO99^A^ electrocompetent cells, which were then cultured at 28 °C with shaking for 6 h. An appropriate volume of the bacterial culture was plated on sucrose-free NA-resistant plates, with a final kanamycin concentration of 50 μg/mL. Following a 3-day incubation at 28 °C, monoclonal colonies were selected and streaked onto new NA-resistant plates, and colony PCR screening was performed to identify single crossovers. Verified single crossovers exhibited two distinct bands: one representing the combined upstream, knockout, and downstream fragments; and the other representing the combined upstream and downstream fragments. These single crossovers were subsequently stored. For the second homologous recombination, high-sucrose NA plates were utilized to select double crossovers. Single crossovers were cultured with shaking for 8 h, and 50 μL of the bacterial suspension was plated onto 10% sucrose NA plates. After a 3-day incubation at 28 °C, monoclonal colonies were streaked onto NA plates, and colony PCR screening was conducted to identify the mutants. Correct mutants showed a single band corresponding to the combined size of the upstream and downstream fragments. These mutants were then preserved. The PCR primers for constructing the mutant strain SK01 are listed in Table S1.

### 4.3. Phenotypic Characterization of Bacterial Growth

#### 4.3.1. Observation of Bacterial Morphology

The mutant and wild-type strains of *X. oryzae* pv. *oryzae*, SK01 and PXO99^A^, were preserved at −80 °C using a 10% glycerol solution. For reactivation, 10 µL of each glycerol-preserved bacteria was streaked onto an NA plate and incubated at 28 °C for 3 days to observe colony growth. Further, 2 µL of bacterial suspension from a liquid NA culture at an OD 600 nm of 1.0 was spotted onto NA plates and observed after incubation at 28 °C for 3 days to assess the growth form.

#### 4.3.2. Measurement of Bacterial Growth Rate

The reactivated strains were grown in an NA liquid medium until they reached an OD 600 nm of 1.0. Subsequently, 10 µL of this culture was transferred to 20 mL of fresh NA medium and incubated at 28 °C with shaking at 200 rpm. The OD 600 nm was recorded starting at the 20th h and every 4 h thereafter until the 40th h. This experiment was conducted with five replicates per group and repeated three times biologically.

#### 4.3.3. Assessment of Bacterial Biofilm Formation

The bacterial strains of *X. oryzae* pv. *oryzae* were cultured in an NA medium until reaching an OD 600 nm of 1.0. The cultures were then transferred at a 1% inoculation rate into glass tubes containing 4 mL of NA medium and incubated with shaking at 28 °C for 2 days, followed by static incubation for an additional 5 days. The bacterial suspension in the tubes was gently pipetted out from the tubes, which were then washed twice with sterile water and subsequently dried at 60 °C. For biofilm staining, 4 mL of 1% crystal violet was added to each dried tube and allowed to stand for 10 min. The excessive staining solution was then carefully removed with a pipette, and the tubes were gently rinsed three times with sterile water and dried at 37 °C. The formation of a purple biofilm at the air–liquid interface in the tubes was observed and photographed. To quantify the biofilms, an equal volume of 95% ethanol was added to each tube, and the contents were mixed thoroughly by pipetting to dissolve the purple substance. The absorbance of the samples was then measured at 590 nm using a spectrophotometer.

### 4.4. Evaluation of Bacterial Pathogenicity

#### 4.4.1. Pathogenicity Testing of Rice

One-month-old rice plants were used for inoculation with *X. oryzae* pv. *oryzae*. For inoculation, sterile scissors were used to dip into the bacterial culture with an OD 600 nm of approximately 0.5), and then, to cut the rice leaves about 2 cm from their tips, allowing the bacterial fluid to penetrate the rice tissues. Two weeks post-inoculation, the lengths of the lesions were measured and subjected to statistical analysis. Each bacterial strain was inoculated into ten plants, with three leaves inoculated per plant.

#### 4.4.2. Quantification of Bacterial Colonization

Three days after inoculation, rice leaves were clipped, cut into small segments, and surface-sterilized by immersion in 75% ethanol for 30 s. After a single rinse with sterile water and drying with sterile cotton balls, the leaf segments were homogenized in a sterile mortar with 1 mL of sterile water. This homogenate was serially diluted from 10^−2^ to 10^−6^, and 50 µL of each dilution was plated on NA plates. After incubating these plates at 28 °C for 3 days, bacterial colony-forming units were counted. The results, depicted in Figure 4, indicated that 2 weeks after inoculation with bacteria, leaves in the mock control group (containing only NA medium) maintained healthy growth while the PXO99^A^-inoculated group exhibited extensive wrinkling and yellow-white lesions along the veins, with lesion lengths exceeding 10 cm, and high bacterial loads inside leaf tissues. Conversely, the leaves of the SK01-inoculated group showed good leaf growth, with markedly reduced lesion lengths and bacterial densities inside the cells, indicating that deletion of the sRNA trans3287 sequence led to a significant loss of pathogenicity in the SK01 strain.

#### 4.4.3. Quantification of the Expression of Key Virulence-Related Genes

Bacterial cultures were grown to an OD 600 nm of 1.0, after which the cells were collected in RNase-free centrifuge tubes. Subsequently, bacterial cultures (4 mL per tube) were centrifuged at 12,000 rpm for 1 min to precipitate the cells, followed by discarding the supernatant. Bacterial RNA was extracted from these bacterial cells using Trizol reagent, and RNA was reverse transcribed following the manufacturer’s protocol of the Vazyme R423 kit. Gene expression was quantified through an RT-qPCR kit (Vazyme Q711). The PCR cycling conditions were set as follows: initial denaturation at 95 °C for 30 s, followed by 40 cycles of denaturation at 95 °C for 5 s, annealing at 60 °C for 30 s, a final extension at 95 °C for 15 s, and a final holding at 60 °C for 1 min. The primers used for quantifying the expression of virulence-related genes are detailed in Table S1.

### 4.5. Construction of Dual-Plasmid Vectors

Recombinant vectors were constructed using homologous recombination. The pXG10SF plasmid was linearized using the NheI restriction enzyme, and the pZK001 plasmid was linearized using the PstI restriction enzyme. Positive colonies were screened and verified using colony PCR, and they were then sequenced for accuracy and stored for future use. The low-copy-number vector pXG10SF, carrying the target gene, and the high-copy-number plasmid pZK001, carrying the sRNA, were co-transformed into *E. coli* TOP10 cells. Correct colonies were confirmed via PCR and cultured in a shaking incubator. The co-transformation of pXG10SF::*PXO_03470* with pZK001::*trans3287* was designated as the experimental group. The co-transformations of pXG10SF::*PXO_03470* with pZK001 and pXG10SF with pZK001::*trans3287* were used as control groups for comparative analyses.

### 4.6. Measurement of the Expression of Defense Response Genes

#### 4.6.1. Plant Total RNA Extraction

Rice leaf tissues (approximately 100 mg) were cut with sterile scissors and immediately ground in a mortar with liquid nitrogen. The resulting tissue powder was transferred to an RNase-free 2 mL centrifuge tube. Trizol reagent (1 mL) was added for thorough lysis, and the mixture was vigorously shaken and incubated on ice for 5 min. Chloroform (200 µL) was added, the mixture was shaken for 15 s, and then, left to stand at room temperature for 10 min. The mixture was centrifuged (12,000× *g*) at 4 °C for 10 min. The upper aqueous phase was carefully transferred to a new centrifuge tube, and the chloroform extraction was repeated. Isopropanol (0.5 mL) was added, and the tube was inverted five times for mixing. The samples were left to stand at 4 °C overnight. After centrifugation, the supernatant was discarded. The RNA pellet was washed with 1 mL of 75% ethanol (prepared with DEPC-treated water), centrifuged, and the supernatant discarded again. The pellet was air-dried with the tube open to ensure complete ethanol evaporation. Finally, the RNA was dissolved in 50 µL of RNase-free ultrapure water and stored at a low temperature for subsequent experiments.

#### 4.6.2. Reverse Transcription of Plant RNA

The extracted RNA was reverse transcribed using the Vazyme R423 kit according to the manufacturer’s protocol.

#### 4.6.3. Quantification of Defense Gene Expression in Plants

The expression of the plant defense gene *OsPR1b* was measured using an RT-qPCR kit (Vazyme Q711), with the specific primers outlined in Table S1. This method provided a precise measure of *OsPR1b* gene expression under experimental conditions.

### 4.7. Protein Interaction Assay

#### 4.7.1. Protein Extraction

*E. coli* cultures were grown to an OD 600 nm of 2.0 and then harvested. Bacterial cells (10 mL) were washed twice with phosphate-buffered saline (PBS) and resuspended in 2 mL of PBS containing 1 mM phenylmethylsulfonyl fluoride (PMSF) for protease inhibition. The suspension was subjected to sonication for 5 min to lyse the cells. Afterward, the lysate was centrifuged at a low temperature to separate the supernatant, which was then mixed with protein loading buffer and boiled for 10 min to denature the proteins.

#### 4.7.2. Sodium Dodecyl Sulfate–Polyacrylamide Gel Electrophoresis (SDS-PAGE) and Protein Transfer

Proteins were separated using 6% SDS-PAGE under initial conditions of 60 V for 30 min, then at 120 V for approximately 2 h. Thereafter, polyvinylidene fluoride (PVDF) membranes and four pieces of filter paper were cut and prepared. The membrane was soaked in methanol solution, and the gel and filter paper were soaked in transfer buffer (containing 0.582 g of Tris, 0.293 g of glycine, 0.375 mL of 10% SDS, 20 mL of chromatography-grade methanol per 100 mL, and water up to 100 mL). A transfer “sandwich” was assembled and placed in a transfer device set to a constant voltage of 25 V for 1 h. The PVDF membrane was then removed and the remainder was discarded.

#### 4.7.3. Immunoblotting

The PVDF membrane was submerged in TBST buffer containing skim milk (12.1 g of Tris, 9 g of NaCl, 500 μL of Tween-20 per 1 L, adjusted to pH 7.5 with HCl, with 0.5 g of skim milk added per 10 mL of TBST) and shaken at room temperature for 1 h. The membrane was incubated with 20 mL of TBST containing skim milk powder and GFP antibody, and then, agitated at room temperature for 3 h. The membrane was washed four times with TBST, each for 5 min. Subsequently, the membrane was incubated with a secondary antibody (appropriate to the primary antibody’s source) in TBST containing skim milk powder and gently agitated for 1 h. The membrane was washed four times with TBST, with each wash lasting 5 min. For color development, 2 mL of the color development solution was prepared, and the membrane was immersed in it for approximately 2 min, then analyzed using an imaging system.

## Figures and Tables

**Table 1:** Predicted targets of *X. oryzae* pv. *oryzae* strain PXO99^A^ sRNA trans3287.

Rank	Synonym	Energy	*p* Value	Predicted Function
1	*PXO_01948*	−11.34	0.011	alpha/beta hydrolase
2	*PXO_01019*	−11.22	0.012	c-di-GMP phosphodiesterase
3	*PXO_03470*	−10.96	0.014	cardiolipin synthase, cls
4	*PXO_00139*	−9.89	0.025	endolysin
5	*PXO_03758*	−9.64	0.028	FMN reductase
6	*PXO_01913*	−8.53	0.045	reductase
7	*PXO_04697*	−8.43	0.047	type VI secretion system-associated protein

## Data Availability

The raw data supporting the conclusions of this article will be made available upon request to the corresponding authors.

## Supplementary Materials

The following supporting information can be downloaded at: https://www.mdpi.com/article/10.3390/plants13182576/s1, Table S1: Information on genes tested and primers used in this study.
