# Supplementary material for: Strain of Xanthomonas oryzae pv. oryzae Loses Virulence through Dysregulation of Cardiolipin Synthase
Source: Plants (Basel). 2024 Sep 14;13(18):2576. doi: 10.3390/plants13182576 (PMC11435070; doi:10.3390/plants13182576)
Supplement: Supplementary file 1 [file plants-13-02576-s001.zip › plants-3104050-supplementary.pdf]

Supplemental Data

Additional File 1: Table S1. Information on genes tested and primers used in this study.

Gene	Primers / subjects
trans3287	Upstream homologous arm:<br>F: 5′-TTTTCGCCTTTCCCTCAC-3′<br>R: 5′-AAGCGCACACGGTGGAT-3′<br>Downstream homologous arm:<br>F: 5′-ATGCAGGTAATTTGTGAGGAC-3′<br>R: 5′-AACCCTTGTCCTTAGTTGCC-3′ / deletion
	F: 5′-CCGTTCGCTCGTCACTACTT-3′<br>R: 5′-ATAAGGGTGAGGTCGGTAGT-3′ / validation first homologous recombination
	F: 5′-TTTTCGCCTTTCCCTCAC-3′<br>R: 5′-AACCCTTGTCCTTAGTTGCC-3′ / validation second homologous recombination
	F: 5′-CTTATCAGGGGTGCGCTCTA-3′<br>R: 5′-TCGGTATGCGAAGTCCCTTT-3′ / RT-qPCR
	F: 5′-CGTGAACCTGCAATTCGCTTGACC-3′<br>R: 5′-ATTTGATGCCTCCTCAGAGATTC-3′ / fusion with pZK001
rpfC	F: 5′-GTTGGCGAACTGGTGGTATC-3′<br>R: 5′-GCACGTAGATAGTTGCTGCC-3′ / RT-qPCR
cysB	F: 5′-CAGCGCATGAAACTGTACGA-3′<br>R: 5′-GATCCAGCTTGCTCACCATG-3′ / RT-qPCR
metB2	F: 5′-GATCTGGTGCTGCATTCCAC-3′<br>R: 5′-AAGGTGAGGAAGGCATCGAA-3′ / RT-qPCR
wxoA	F: 5′-CGAGCTGTTTACGGTGAAGG-3′<br>R: 5′-CACACCACGCAGCAAGAATA-3′ / RT-qPCR
wxoB	F: 5′-GTGCAGTGGTGTTGGTCAAT-3′<br>R: 5′-TCGAGGGTCATCACTTGCTT-3′ / RT-qPCR
wxoD	F: 5′-CTTGGGTGGTTATGGGGTCT-3′<br>R: 5′-TAACAGCTGGCCCGAGTAAA-3′ / RT-qPCR
xadA	F: 5′-AATCCTTGGCCAGTGGTGAA-3′<br>R: 5′-GCCGCTACATTGGTGATCTG-3′ / RT-qPCR
xadB	F: 5′-CTATGCCTTGGCATCGAACC-3′<br>R: 5′-AGGCATCGTCTACCCAGTTG-3′ / RT-qPCR
hrpG	F: 5′-CGCAATGTCTCGGTGTTC-3′<br>R: 5′-GCTGAGTTGCTGCGTTTCC-3′ / RT-qPCR
PXO_01948	F: 5′-GCTTTTGAAGTATCGCGTGC-3′<br>R: 5′-AAATACGTACTGATCGCGGC-3′ / RT-qPCR

PXO_01019	F: 5′-CCGCTGATGATTTTCCACGT-3′<br>R: 5′-CAGTTGCTGGCGGATTTCTT-3′ / RT-qPCR
PXO_03470	F: 5′-ATCTTCCAGCCCGACCATAG-3′<br>R: 5′-CAATTGCGAGGGATGGAACC-3′ / RT-qPCR
	F: 5′-ACCGCCTCGCTATGCTCGCATTTG-3′<br>R: 5′-CAGCGGATCCGCTGAGATTCATACC-3′ / fusion with pXG10SF
PXO_00139	F: 5′-CGCATCCAAGAAGCCATCG-3′<br>R: 5′-TTGCGCGTCCATCTTGATTC-3′ / RT-qPCR
PXO_03758	F: 5′-TTCGGCCGCTTTTCAGTTTT-3′<br>R: 5′-GTTCTGTGCGGTGATGGAAC-3′ / RT-qPCR
PXO_01913	F: 5′-GATGGATCGTTTGTTCGGCA-3′<br>R: 5′-ACCAGCTTGAACAAACTGCG-3′ / RT-qPCR
PXO_04697	F：5′-GACATCCACTACGCGTTCCT-3′<br>R：5′-CAGACCTTGGACATCGCAAG-3′ / RT-qPCR
